# A real-world pilot study assessing treatment satisfaction with avanafil in patients with erectile dysfunction

**DOI:** 10.1093/sexmed/qfae001

**Published:** 2024-02-07

**Authors:** Ping-Ju Tsai, Shih-Ya Hung, Tsung-Hsi Lee, Bang-Ping Jiann

**Affiliations:** Division of Urology, Department of Surgery, Yuan's General Hospital, No. 136, Siwei 4th Road, Lingya District, Kaohsiung City 802793, Taiwan, Republic of China; Division of Urology, Department of Surgery, Yuan's General Hospital, No. 136, Siwei 4th Road, Lingya District, Kaohsiung City 802793, Taiwan, Republic of China; Division of Urology, Department of Surgery, Yuan's General Hospital, No. 136, Siwei 4th Road, Lingya District, Kaohsiung City 802793, Taiwan, Republic of China; Division of Urology, Department of Surgery, Yuan's General Hospital, No. 136, Siwei 4th Road, Lingya District, Kaohsiung City 802793, Taiwan, Republic of China

**Keywords:** avanafil, phosphodiesterase type 5 inhibitor, erectile dysfunction, treatment satisfaction

## Abstract

**Background:**

Avanafil is a second-generation phosphodiesterase type 5 (PDE5) inhibitor, and offers a rapid onset of action (15 minutes). Its real-world data, including treatment satisfaction, are still lacking.

**Aim:**

The study sought to investigate the treatment outcomes of avanafil and the factors impacting treatment satisfaction in a real-world setting.

**Methods:**

Between November 2021 and February 2023, erectile dysfunction (ED) patients prescribed avanafil were consecutively enrolled in this phase 4, open-label, cross-sectional, observational study. At each follow-up visit (4-week intervals), participants completed a questionnaire for assessing the use and treatment-emergent adverse events of avanafil, ED severity, and treatment satisfaction.

**Outcomes:**

The outcome measures included the Sexual Health Inventory for Men (SHIM), and Erectile Dysfunction Inventory of Treatment Satisfaction.

**Results:**

Among 234 patients enrolled, 112 (47.9%) patients had follow-up visits and answered the questionnaire. Treatment with avanafil significantly improved the mean SHIM total score from 10.2 ± 5.6 at baseline to 17.5 ± 6.2 (*P* < .001). Of the patients treated with avanafil, 71.4% (n = 80 of 112) reported a >4-point improvement in the SHIM total score, and 33.1% (n = 37 of 112) reported normal erectile function. The proportion of patients satisfied with avanafil treatment (defined as Erectile Dysfunction Inventory of Treatment Satisfaction index score ≥60) was 87.5%. Several physical factors (younger age, lower waist circumference, and lower level of low-density lipoprotein), and sexual function factors (shorter duration of ED, higher SHIM total score at baseline, PDE5 inhibitor treatment naive, and acquired premature ejaculation) tended to contribute to satisfaction with avanafil treatment. Treatment-emergent adverse events occurred in 41.1% of patients, and all were mild in severity.

**Clinical Implications:**

This study identifies the factors associated with treatment satisfaction of avanafil, which may ultimately lead to better treatment outcomes.

**Strengths and Limitations:**

This is the first study to provide real-world evidence of avanafil for ED treatment, and validated questionnaires were used to assess erectile function and treatment satisfaction. However, the limitations of this study include single-center observational study design, small sample size, and short-term follow-up.

**Conclusion:**

Avanafil is an effective treatment for ED, and satisfaction rate is high in an outpatient setting. The awareness of identified factors related to patient satisfaction may improve treatment outcomes.

## Introduction

Erectile dysfunction (ED) is defined as the inability of the male to attain and maintain erection of the penis sufficient to permit satisfactory sexual intercourse.^1^ The global prevalence of ED is 3% to 76.5%, and there is a trend of increasing ED prevalence with increasing age (10.6%-35.0% in men 18-40 years of age, 15.5%-65.4% in men 40-70 years of age, and 27.7%-94.9% in men aged ≥70 years of age).^2^ According to main underlying disorders, ED can be classified into organic, psychogenic and mixed, and mixed ED is the most common type.^3^ Risk factors for ED include age, diabetes mellitus, dyslipidemia, hypertension, cardiovascular disease, premature ejaculation (PE), benign prostatic hyperplasia, obesity/waist circumference, smoking, and anxiety.^3^ ED has negative effects on psychosocial health and quality of life and is a risk marker for cardiovascular disease and mortality.^2^^,^^3^

The treatment goals of ED should be individualized to restore sexual satisfaction for the patient/couple and improve quality of life based on the patient’s expressed needs and desires.^4^ Treatment satisfaction in ED patients can be affected by multiple factors, including the quality of erection, unacceptable adverse events, spontaneity, couple’s expectations, and cost.^4^ A better understanding of factors with significant impact on treatment satisfaction can help tailor future treatment strategies and improve treatment outcomes.

ED may be associated with modifiable or reversible risk factors, and lifestyle changes and risk factor modifications can be initiated either before or at the same time as ED treatments are used.^3^ Psychosocial interventions/therapies, such as sexual skills training, psychosexual education, and cognitive behavioral therapy, allow the management of psychosocial factors contributing to ED, and combining of cognitive behavioral therapy with medical treatment for ED can maximize treatment outcomes.^3^ First-line treatment options for ED include oral phosphodiesterase type 5 (PDE5) inhibitors, intracavernous injections therapy, topical/intraurethral alprostadil, vacuum erection devices, and low-intensity shockwave therapy.^3^ The surgical implantation of a penile prosthesis is typically reserved as the last-line treatment option.^3^

Oral PDE5 inhibitors, including sildenafil, tadalafil, vardenafil, and avanafil, are the most common first-line therapy for ED.^3^^,^^5^ Avanafil, a second-generation PDE5 inhibitor, is designed to be fast-acting and highly selective.^6-8^ Avanafil has a rapid onset of action (as early as 15 minutes), which eliminates the inconvenience of patients having to wait a considerable time before attempting intercourse.^9^^,^^10^ The duration of effect of avanafil may maintain beyond 6 hours after dosing.^9^

Avanafil have been shown to be an effective agent for ED treatment with well tolerance in clinical trials^9^^,^^11-13^; however, the factors related to treatment satisfaction with avanafil have not been explored. In the present study, we aimed to address 2 questions: (1) Is avanafil effective in a real-world practice setting? and (2) Do outpatients with ED report high rate of satisfaction with avanafil treatment? Therefore, we conducted a cross-sectional observation study to investigate its real-world effectiveness and treatment satisfaction assessed by a validated questionnaire.

## Methods

### Study design and study participants

In this phase 4, open-label, cross-sectional, observational study, ED patients were consecutively recruited from our outpatient clinic between November 2021 and February 2023. At the baseline visit (visit 1), a complete medical history was obtained and a physical examination was performed. Hypogonadism was defined as serum total testosterone <350 ng/dL. All participants were instructed to complete the Sexual Health Inventory for Men (SHIM) questionnaire to evaluate erectile function over the past 6 months.^14^ They were prescribed 2 boxes of avanafil (4 × 200 mg tablets per box) for on-demand use and were recommended to take avanafil ≥15 minutes before sexual activity, with sexual stimulation to facilitate an erection. Posttreatment data, including treatment-emergent adverse events (TEAEs), erectile function, and treatment satisfaction, were collected using a questionnaire at each follow-up visit (4-week intervals).

The study protocol was reviewed and approved by the Institutional Review Board at our institution. A waiver of written informed consent was approved by the Institutional Review Board because the present study was conducted using a retrospective chart review method. However, the purpose of this study had been well explained to all participants before answering the questionnaire.

Subjects who were willing to purchase avanafil for ED treatment were enrolled into this study. Patients who had a history of prostate cancer, major psychiatric disease, debilitating diseases, or unstable cardiopulmonary function; received any form of nitroglycerin therapy; or had sex with men were excluded from the study.

### Outcome measures

At each follow-up visit (visits 2-6), a structured questionnaire consisting of 21 questions was utilized to enquire the clinical use of avanafil. It was divided into 3 sections:

1) Section 1 contained 5 questions about the dose, administration time, TEAE checklist, and TEAE severity of avanafil, and previous history of ED treatment with PDE5 inhibitors.
2) Section 2 was the 5-item SHIM questionnaire.^14^ Due to short-term treatment period in this study, the SHIM questionnaire was used to measure erection function over the past 4 weeks in participants at follow-up visits, rather than over the past 6 months (original version). The severity of ED was classified into 5 categories based on the SHIM total score: severe ED (1-7), moderate ED (8-11), mild-to-moderate ED (12-16), mild ED (17-21), and no ED (22-25).
3) Section 3 was the patient version of 11-item Erectile Dysfunction Inventory of Treatment Satisfaction (EDITS) questionnaire.^15^ Each question was scored on a 5-point scale, with a higher score indicating greater satisfaction. An EDITS index score was calculated by multiplying EDITS total score by 1.82, resulting in a range from 20 (lowest satisfaction) to 100 (highest satisfaction). Treatment satisfaction was defined as an EDITS index score ≥60 and treatment dissatisfaction was defined as an EDITS index score <60.

With the exception of the SHIM and EDITS, which are validated questionnaires, the other questions have not been validated.

### Statistical analysis

Data were reported as mean ± SD or number of patients and percentage. Categorical variables were compared by chi-square test. Continuous variables were analyzed by unpaired Student’s *t* test.

Excel 2019 (Microsoft) was used for data entry, and statistical analysis was performed using Stata version 17.0 (StataCorp). The null hypothesis was rejected when a *P* value was <.05.

## Results

### Baseline characteristics

A total of 234 patients were included in this study, and their baseline characteristics are summarized in Table 1. The mean age of study population was 54.6 ± 11.9 years (range, 22-82 years). Nearly half of patients (46.6%) were current or former smokers. Many participants had concomitant diseases, and the commonly reported comorbidities (>20%) were hypogonadism (40.7%), dyslipidemia (36.8%), hypertension (31.6%), PE (27.0%), and diabetes mellitus (20.9%). The mean duration of ED was 2.6 ± 3.2 years (range, 0.3-20.0 years). At the baseline visit, the mean SHIM total score was 10.6 ± 5.7, indicating moderate severity of ED on average.

**Table 1:** Baseline demographic and laboratory data of 234 patients.

Variable	No. of patients with data	Result
Age, y	234	54.6 ± 11.9
Waist circumference, cm	234	116.1 ± 11.0
Body mass index, kg/m^2^	233	25.9 ± 3.3
Smoking habit	234	
Never smoker		125 (53.4)
Current smoker		51 (21.8)
Former smoker		58 (24.8)
Diabetes mellitus	234	49 (20.9)
Hypertension	234	74 (31.6)
Dyslipidemia	234	86 (36.8)
MACE	234	16 (6.8)
Total testosterone, mg/dL	189	4.3 ± 2.1
Hypogonadism	189	77 (40.7)
Cholesterol, mg/dL	181	179.6 ± 42.6
Triglyceride, mg/dL	184	144.1 ± 81.5
HDL, mg/dL	176	48.0 ± 18.4
LDL, mg/dL	179	111.7 ± 33.8
FBG, mg/dL	171	111.0 ± 35.5
HbA1c, %	107	6.6 ± 1.3
ED duration, y	228	2.6 ± 3.2
SHIM total score	234	10.6 ± 5.7
ED severity	234	
Severe		79 (33.8)
Moderate		56 (23.9)
Mild to moderate		60 (25.6)
Mild		32 (13.7)
Normal		7 (3.0)
Coexisting PE	233	63 (27.0)
Lifelong PE		6 (2.6)
Acquired PE		57 (24.5)

Values are n, mean ± SD, or n (%).

Abbreviations: ED, erectile dysfunction; FBG, fasting blood glucose; HbA1c, glycated hemoglobin; HDL, high-density lipoprotein; LDL, low-density lipoprotein; MACE, major adverse cardiovascular events; PE, premature ejaculation; SHIM, Sexual Health Inventory for Men.

### Factors associated with follow-up visits

During the study period, 112 (47.9%) patients had follow-up visits. The results of analyses for the correlation of baseline characteristics with follow-up visits indicated that patients with follow-up visits had a lower level of fasting blood glucose (FBG) (105.7 ± 26.8 mg/dL vs 116.7 ± 42.4 mg/dL; *P* = .043) and purchased more boxes of avanafil at visit 1 (2.1 ± 0.6 boxes per patient vs 2.0 ± 0.4 boxes per patient; *P* = .028) than patients without follow-up visits.

### Effectiveness of avanafil

The questionnaire was completed by 112 patients (response rate: 47.9%), and the mean follow-up duration in these patients was 5.6 ± 3.1 weeks (range, 4-20 weeks). The proportions of patients receiving 100 mg, 200 mg, and >200 mg of avanafil were 13.4%, 82.1%, and 4.5%, respectively. The majority of patients (94.6%) took avanafil <2 hours before sexual activity (<15 minutes for 17.0%, 15-60 minutes for 58.9%, and 1-2 hours for 18.8%).

The mean SHIM total score significantly increased from 10.2 ± 5.6 at baseline to 17.5 ± 6.2 after avanafil treatment (*P* < .001) (Table 2). A >4-point increase in the SHIM total score was achieved by 71.4% of patients, and 33.1% of patients had normal erectile function as a result of treatment with avanafil.

**Table 2:** Change in erectile function after avanafil treatment in 112 patients with follow-up visits.

Variable	Baseline	After avanafil treatment
SHIM total score	10.2 ± 5.6	17.5 ± 6.2^a^
ED severity		
Severe	43 (38.4)	9 (8.0)
Moderate	28 (25.0)	11 (9.8)
Mild to moderate	26 (23.2)	24 (21.4)
Mild	11 (9.8)	31 (27.7)
Normal	4 (3.6)	37 (33.1)
Patients reporting a >4-point increase in the SHIM total score after avanafil treatment	**—**	80 (71.4)

Values are mean ± SD or n (%).

Abbreviations: ED, erectile dysfunction; SHIM, Sexual Health Inventory for Men.

a Significant difference vs baseline.

**Figure 1:**
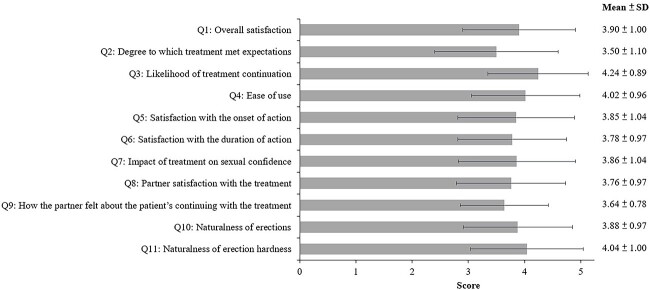
Results of the Erectile Dysfunction Inventory of Treatment Satisfaction (EDITS) in 112 patients with follow-up visits. Data are presented as mean ± SD.

Avanafil treatment resulted in the mean scores for 11 EDITS items ranging from 3.50 (question 2: Degree to which treatment met expectations) to 4.24 (question 3: Likelihood of treatment continuation) (Figure 1). The mean EDITS index score was 77.22 ± 15.91, and the satisfaction rate of avanafil treatment (the proportion of patients with an EDITS index score ≥60) was as high as 87.5%.

### Factors associated with treatment satisfaction

While 98 patients were satisfied with avanafil treatment (mean EDITS index score = 81.2 ± 12.4), 14 patients were not satisfied (mean EDITS index score = 49.6 ± 8.5) (Table 3). Younger age, lower waist circumference, and lower level of low-density lipoprotein (LDL) were associated with satisfaction with avanafil treatment. As to male sexual function, patients reporting satisfaction with avanafil treatment had a shorter duration of ED (2.3 ± 3.2 years vs 4.4 ± 3.7 years; *P* = .029), higher SHIM total score at baseline (10.6 ± 5.4 vs 7.1 ± 5.6; *P* = .025) and after avanafil treatment (18.8 ± 5.0 vs 8.3 ± 5.6; *P* < .001), and a higher proportion of PDE5 inhibitor–naïve men (52.0% vs 14.3%; *P* = .008) than patients reporting dissatisfaction with avanafil treatment. Although the proportions of patients with PE were similar between the 2 subgroups (23.7% vs 28.6%; *P* = .692), PE type was a significant factor affecting treatment satisfaction with avanafil (*P* = .015). In the satisfaction subgroup, the proportion of patients with lifelong PE was lower (1.0% vs 14.3%), but the proportion of patients with acquired PE was higher (22.7% vs 14.3%) than in the dissatisfaction subgroup.

**Table 3:** Comparison of baseline characteristics and treatment outcomes between patients reporting satisfaction (EDITS index score ≥60) and patients reporting dissatisfaction (EDITS index score <60) with avanafil treatment.

Variable	Patients reporting satisfaction with avanafil treatment (n = 98)	Patients reporting dissatisfaction with avanafil treatment (n = 14)	*P* value
Age, y	54.0 ± 11.2	60.6 ± 10.5	.041^a^
Waist circumference, cm	116.2 ± 10.3	122.8 ± 15.2	.038^a^
Body mass index, kg/m^2^	26.1 ± 3.4	25.7 ± 2.8	.649
Smoking habit			.346
Never smoker	57 (58.2)	6 (42.9)	
Current smoker	17 (17.3)	2 (14.3)	
Former smoker	24 (24.5)	6 (42.9)	
Diabetes mellitus	18 (18.4)	3 (21.4)	.784
Hypertension	32 (32.7)	5 (35.7)	.820
Dyslipidemia	38 (38.8)	3 (21.4)	.208
MACE	6 (6.1)	1 (7.1)	.511
Total testosterone, mg/dL	4.4 ± 2.1	4.3 ± 2.2	.834
Hypogonadism	34 (35.1)	5 (35.7)	.961
Cholesterol, mg/dL	172.3 ± 42.7	190.6 ± 32.2	.174
Triglyceride, mg/dL	149.3 ± 95.8	127.7 ± 50.3	.467
HDL, mg/dL	49.0 ± 24.2	46.1 ± 10.9	.707
LDL, mg/dL	105.9 ± 32.0	128.9 ± 26.1	.025^a^
FBG, mg/dL	106.9 ± 27.2	97.2 ± 10.8	.261
HbA1c, %	6.4 ± 1.1	6.2 ± 1.2	.610
ED duration, y	2.3 ± 3.2	4.4 ± 3.7	.029^a^
SHIM total score (baseline)	10.6 ± 5.4	7.1 ± 5.6	.025^a^
ED severity (baseline)			.595
Severe	35 (35.7)	8 (57.1)	
Moderate	25 (25.5)	3 (21.4)	
Mild to moderate	24 (24.5)	2 (14.3)	
Mild	10 (10.2)	1 (7.1)	
Normal	4 (4.1)	0 (0.0)	
SHIM total score (posttreatment)	18.8 ± 5.0	8.3 ± 5.6	<.001^a^
ED severity (posttreatment)			<.001^a^
Severe	3 (3.1)	6 (42.9)	
Moderate	6 (6.1)	5 (35.7)	
Mild to moderate	22 (22.4)	2 (14.3)	
Mild	31 (31.6)	0 (0.0)	
Normal	36 (36.7)	1 (7.1)	
Patients reporting a >4-point increase in the SHIM total score after avanafil treatment	76 (77.6)	4 (28.6)	<.001^a^
Coexisting PE	23 (23.7)	4 (28.6)	.692
Lifelong PE	1 (1.0)	2 (14.3)	.015^a^
Acquired PE	22 (22.7)	2 (14.3)	
Purchase of avanafil per patient at visit 1, boxes	2.10 ± 0.62	2.14 ± 0.66	.865
Total purchases of avanafil per patient, boxes	3.90 ± 2.92	3.86 ± 3.72	.963
Previous ED treatment			
PDE5 inhibitor naïve	51 (52.0)	2 (14.3)	.008^a^
PDE5 inhibitor pretreated	47 (48.0)	12 (85.7)	.008^a^
EDITS index score	81.2 ± 12.4	49.6 ± 8.5	<.001^a^
Patients reporting any TEAE	41 (42.3)	5 (35.7)	.642
Follow-up duration, wk	5.6 ± 3.2	5.7 ± 3.1	.947

Values are mean ± SD or n (%).

Abbreviations: ED, erectile dysfunction; EDITS, Erectile Dysfunction Inventory of Treatment Satisfaction; FBG, fasting blood glucose; HbA1c, glycated hemoglobin; HDL, high-density lipoprotein; LDL, low-density lipoprotein; MACE, major adverse cardiovascular event; PDE5, phosphodiesterase type 5; PE, premature ejaculation; SHIM, Sexual Health Inventory for Men; TEAE, treatment-emergent adverse event.

a Significant difference.

### Impact of previous PDE5 inhibitor therapy

Among 112 responders to the questionnaire, 53 (47.3%) were naïve to PDE5 inhibitor therapy and 59 (52.7%) had used other PDE5 inhibitors than avanafil. Subgroup analyses demonstrated that a higher proportion of patients in the PDE5 inhibitor–naïve subgroup were satisfied with avanafil treatment than those in the PDE5 inhibitor–pretreated subgroup (96.2% vs 79.7%; *P* = .008) (Table 4). However, no differences in the proportions of patients reporting a >4-point increase in the SHIM total score and any TEAE after avanafil treatment and follow-up duration between the 2 subgroups were observed.

**Table 4:** Comparison of treatment outcomes between PDE5 inhibitor–naïve patients and PDE5 inhibitor–pretreated patients.

Variable	PDE5 inhibitor–naïve patients (n = 53)	PDE5 inhibitor–pretreated patients (n = 59)	*P* value
Patients reporting satisfaction with avanafil treatment (EDITS index score ≥60)	51 (96.2)	47 (79.7)	.008^a^
Patients reporting a >4-point increase in the SHIM total score after avanafil treatment	38 (71.7)	42 (71.2)	.952
Patients reporting any TEAE	22 (41.5)	24 (41.4)	.989
Follow-up duration, wk	5.7 ± 3.5	5.6 ± 2.7	.877

Values are n (%) or mean ± SD.

Abbreviations: EDITS, Erectile Dysfunction Inventory of Treatment Satisfaction; PDE5, phosphodiesterase type 5; SHIM, Sexual Health Inventory for Men; TEAE, treatment-emergent adverse event.

a Significant difference.

### Safety data

Forty-six (41.1%) patients experienced 1 or more TEAEs. Flushing (21.4%) was the most common TEAE, followed by headache (14.3%) and nasal stuffiness (11.6%). All TEAEs were mild in severity and did not affect the patient’s daily life.

## Discussion

Our study presents real-life experience of the effectiveness and safety of avanafil and the factors influencing follow-up visits and treatment satisfaction. Understanding such factors would be critical to achieve treatment optimization with avanafil in patients with ED.

Follow-up visits are essential to solve treatment problems, such as dissatisfaction with treatment effects, TEAEs, and partner’s acceptance, and to provide continuing education for patients and their partners. However, 52.1% of patients did not attend visit 2 in the present study. In a retrospective study conducted in Taiwan, sildenafil responders had significantly more purchase visits than sildenafil nonresponders (mean 3.26 vs 1.41).^16^ Improvement in response to avanafil therapy might facilitate adherence to follow-up visits.

Higher FBG level and the purchase of fewer boxes of avanafil at visit 1 were found to be significantly correlated with the patient being lost to follow-up after 4 weeks in this study. The mean FBG level in our patients without any follow-up visit was 116.7 mg/dL. In an Italian longitudinal study, elevated FBG (110-125 mg/dL) was associated with higher risks of severe ED, reduced penile blood flow, and overt hypogonadism in male patients with sexual dysfunction.^17^ Response to avanafil might be limited in ED patients with elevated FBG, and risk factor modification should be part of therapeutic strategy in order to achieve effective management of ED in this patient subgroup. McCullough et al^18^ reported that the cumulative probability of achieving intercourse success with sildenafil increased with the number of attempts, and reached a plateau after approximately 8 attempts. The proportion of patients without any follow-up visit among the purchasers of 1 box of avanafil at visit 1 (66.7%) was higher than those among the purchasers of 2 boxes of avanafil (52.5%) and the purchasers of 3 to 6 boxes of avanafil (33.3%). Therefore, patients should be informed that an adequate trial involves ≥6 attempts with avanafil.^3^

In this study, the mean SHIM total score significantly improved from 10.2 to 17.5, and 33.1% of patients had a SHIM total score ≥22 (normal erectile function) after avanafil treatment. Similarly, a significant increase in the mean SHIM total score from 12.4 to 19.9 was observed in Taiwanese patients receiving PDE5 inhibitor therapy, and erectile function returned to normal in 33.3% of patients in our previous observational study.^19^ The proportions of patients with normal erectile function (International Index of Erectile Function erectile function domain score ≥26) after 12-week treatment with avanafil, sildenafil, tadalafil, and vardenafil were 39% to 65%,^20^^-^^22^ 37% to 60%,^22^^,^^23^ 36% to 64%,^24^^,^^25^ and 32% to 56%,^26^^,^^27^ respectively. Higher dose (100/200 mg), longer treatment duration, and less severe ED at baseline were associated with better improvement in erectile function in patients treated with avanafil.^11^^,^^22^

Our patients receiving avanafil reported similar high treatment satisfaction on 11 individual EDITS items, but the mean score for question 9 (How the partner felt about the patient’s continuing with the treatment by patient) was significantly lower than those for the other 10 questions in patients treated with other 3 PDE5 inhibitors.^19^ In this study, the mean EDITS index score was 77.22, which was comparable with the findings from short-term (8-12 weeks) randomized controlled trials (RCTs) of sildenafil (66.5-78.3)^28-30^ and tadalafil (66.8-77).^24^^,^^31^^,^^32^ The data from the present study showed that 87.5% of patients were satisfied with avanafil treatment. High levels of treatment satisfaction (the proportions of patients with an EDITS index score ≥50) with sildenafil were reported in a pooled analysis of phase 2, 3, and 4 clinical trials (86%),^33^ and in a 6-month prospective observational study (89%).^34^

Previous studies of PDE5 inhibitors demonstrated that patient satisfaction (EDITS index score) was correlated significantly with efficacy (SHIM total score), psychosocial benefit (Self-Esteem And Relationship score), and partner satisfaction (EDITS partner index score).^19^^,^^35^^,^^36^ The proportions of patients satisfied with PDE5 inhibitor therapy were higher than the proportions of patients regaining erectile function after PDE5 inhibitor therapy in a Taiwanese observational study (95.5% vs 33.3%)^19^ and a Canadian observational study (89% vs 39%).^34^ A similar trend was found in this study. These data suggested that effectiveness was not the sole determinant of treatment satisfaction in ED patients, and avanafil treatment at least partially met patient expectations.

Montorsi et al^33^ reported that several baseline characteristics, including younger age (≤60 years), less severe ED at baseline, and shorter duration of ED (≤2 years), were related to higher (≥88%) treatment satisfaction rates with sildenafil. In the DETECT study, better erectile function at 12 months was a significant factor associated with higher EDITS index score at 12 months in patients receiving tadalafil treatment.^37^ Our findings were in line with these 2 previous studies.^33^^,^^37^ Furthermore, our patients satisfied with avanafil treatment also tended to have lower waist circumference, lower level of LDL, acquired PE (as compared with lifelong PE), and no previous PDE5 inhibitor treatment.

In the present study, no significant differences in the effectiveness and incidence of any TEAE of avanafil and follow-up duration were observed between PDE5 inhibitor–naïve patients and PDE5 inhibitor–pretreated patients. Previous RCTs also revealed no significant effects of previous PDE5 inhibitor use on the efficacy and safety of avanafil.^20^^,^^21^ Although treatment satisfaction rate with avanafil was significantly higher in PDE5 inhibitor–naïve patients (96.2%) than that in PDE5 inhibitor–pretreated patients (79.7%), high patient satisfaction was observed in both patient subgroups in our study. PDE5 inhibitor–pretreated patients might expect avanafil to provide a similar or better benefit than other PDE5 inhibitors. Therefore, every ED patient has a reasonable chance to be treated successfully with avanafil regardless of previous treatment experience with other PDE5 inhibitors.

The incidence of any TEAE reported here (41.1%) was similar to that observed in previous RCTs of avanafil (100 mg: 10.0%-51.5%; 200 mg: 12.5%-43.7%).^9-11^^,^^20^^,^^21^^,^^38^ Avanafil was well tolerated in our patients with all TEAEs being mild and transient in nature. The most frequently reported TEAEs in this study were flushing, headache, and nasal stuffiness, and were generally consistent with those observed with PDE5 inhibitors.^3^ These TEAEs are related to mild vasodilatory effects of PDE5 inhibitors.^39^ However, the incidence rates of flushing (21.4%), headache (14.3%), and nasal stuffiness (11.6%) in the present study were slightly higher than those in previous RCTs of avanafil 100/200 mg (flushing: 0.0%-13.0%; headache: 0.0%-10.1%; nasal stuffiness: 0.0%-4.3%).^9-11^^,^^20-22^^,^^38^ The use of a TEAE checklist in our study might result in relatively higher reporting rates for TEAEs.

Several limitations existed in this study. As a single-center, observational study, the number of our participants was low, and the monitoring of follow-up was less stringent than that in a RCT. A small sample size might lead to biased estimates and low statistical power. Nearly half (52.1%) of enrolled patients were lost to follow-up after 4 weeks, and their treatment results were not available. Nonresponse bias occurred in this study, and patients without any follow-up visit might be dissatisfied with avanafil treatment. Because patients had to pay for their treatment, the patients with follow-up visits and answering the questionnaire might have higher economic status. The treatment duration of most patients was short (≤4 weeks). Because longer treatment duration may enhance treatment response, our results might be less representative of prolonged avanafil treatment.

## Conclusion

In daily clinical practice, avanafil demonstrated good effectiveness and safety for the treatment of ED. Patient satisfaction with avanafil treatment was high, and was affected by several baseline characteristics and posttreatment erectile function. Our data confirm that avanafil is an appropriate treatment option for ED patients who desire rapid onset of action and on-demand therapy.
